# New Onset of Giant Cell Arteritis following ChAdOx1-S (Vaxevria^®^) Vaccine Administration

**DOI:** 10.3390/vaccines11020434

**Published:** 2023-02-13

**Authors:** Luca Lo Sardo, Simone Parisi, Maria Chiara Ditto, Rosanna De Giovanni, Francesca Maletta, Serena Grimaldi, Luisa Brussino, Enrico Fusaro

**Affiliations:** 1 Unit of Rheumatology, “AOU Città della Salute e della Scienza” Hospital, 10126 Turin, Italy; 2 Clinical Immunology and Allergy Unit, AO Ordine Mauriziano, 10128 Turin, Italy; 3 Pathology Unit, “AOU Città della Salute e della Scienza” Hospital, 10126 Turin, Italy; 4 Division of Nuclear Medicine, “AOU Città della Salute e della Scienza” Hospital, 10126 Turin, Italy

**Keywords:** giant cell arteritis, vaccines, COVID-19, adverse events

## Abstract

We report a 78-year-old man presenting with persistent headaches in vertex and temporo-parietal area; fatigue, worsening after walking; jaw claudication; scotomas; pharyngodynia; and dry cough after the second dose of the SARS-CoV-2 vaccine (ChAdOx1-S) administration. Laboratory findings showed an elevated C-reactive protein level and FDG-CT PET showed evidence of active large vessel vasculitis with diffuse abnormal artery uptake. Under suspicion of vasculitis, a temporal arteries biopsy was performed; the histopathologic findings demonstrated the transmural inflammatory infiltrate with giant cells, compatible with giant cell arteritis. Although the overall incidence of vaccine-triggered autoimmunity is low, rheumatologists worldwide should be aware of autoimmune diseases as a new potential adverse event of vaccines.

## 1. Introduction

Adverse events reported after COVID-19 vaccines usually include injection site reactions and mild systemic symptoms, such as chills, fever, arthralgia, myalgia and headache. Nevertheless, during this vaccination campaign rheumatic diseases arising after vaccines administration have also been described [1]. Moreover, several cases of cutaneous immune complex vasculitis onset after SARS-CoV-2 vaccination have been reported [2,3].

Giant cell arteritis (GCA), also known as Horton’s disease, is a large and medium-sized vessels vasculitis, because it may involve the aorta and great vessels. GCA never occurs before 50 years of age and the major factor risk is aging. The onset of symptoms in GCA tends to be subacute but rarely may begin abruptly. Patients may develop systemic symptoms, such as fever, fatigue, weight loss, headache, jaw claudication and visual involvement. Visual symptoms include diplopia, scotomas, ptosis, blurred vision and loss of vision; brief periods of amaurosis fugax, partial or complete vision loss, in one eye may be rapidly succeeded by permanent irreversible loss of vision. Frequently, GCA is associated with symptoms of polymyalgia rheumatica (PMR). In addition to physical examination, laboratory findings are useful in the assessment of GCA, because many patients have high levels of erythrocyte sedimentation rate and C-reactive protein. A suspected diagnosis of giant cell arteritis should be confirmed by temporal artery biopsy or a temporal artery color Doppler ultrasound. The differential diagnosis of GCA includes other vasculitis (e.g., Takayasu arteritis, medium-sized vessel vasculitis, non-arthritic anterior ischemic optic neuropathy (NAAION) and infections.

The pathogenesis of GCA is incompletely understood, even if a pathophysiological role of infectious agents in great vessel vasculitis, particularly in GCA, has been hypothesized. This conjecture is partly related to the incidence of the disease that has a seasonality and from several reports regarding viruses such as varicella-zoster and, more recently, SARS-CoV-2 as possible triggers of GCA [4]. Similarly, a relationship with the influenza vaccine has been described [5,6]. In a recently published GCA case report related to the SARS-CoV-2 mRNA vaccine, it has been observed that these vaccines can induce cross-reactivity and trigger self-recognition using different mechanisms [7,8].

## 2. Case Report

We report a case of a 78-year-old man with an unusual giant cell arteritis (GCA) onset, after the administration of COVID-19 vaccination. In previous clinical history, the patient reported an exeresis of melanoma, prior Hepatitis B infection and arterial hypertension.

In June 2021, one day after the second dose of SARS-CoV-2 vaccine (ChAdOx1-S), the patient developed a persistent headache in the vertex and temporo-parietal area; fatigue, which worsened after walking; jaw claudication; scotomas; pharyngodynia; and a dry cough not responding to non-steroidal anti-inflammatory drugs. The patient had no symptoms of polymyalgia rheumatica nor visual loss.

No reactions occurred after the first dose of SARS-CoV-2 vaccine (ChAdOx1-S), administered 12 weeks previously. Moreover, he did not report injection site reactions or mild systemic symptoms.

He presented to the general practitioner outpatient clinic who requested a complete blood count, C-reactive protein (CRP), uroculture and serum creatinine. Laboratory tests highlighted normocytic anemia (10.2 g/dl) without leukocytosis, elevated CRP (83.9 g/L), serum creatinine values of 1.13 mg/dl and urine culture negativity. A molecular SARS-CoV-2 test resulted negative, as did a chest X-ray. At the brain and paranasal sinus computed tomography (CT) there was no evidence of ischemic hemorrhagic lesions or bone injuries.

The patient underwent ENT examination with evidence of the mild hyperemia of glottal plan, suggesting the presence of gastroesophageal reflux. Furthermore, the patient underwent a neurological evaluation that evidenced no meningeal irritation signs and no alterations of balance and coordination, mental status, reflexes, nerves functioning or motor and sensory skills assessments; at the temporal arteries examination there was no evidence of tenderness or pain palpations nor a decreased pulse amplitude.

**Figure 1:**
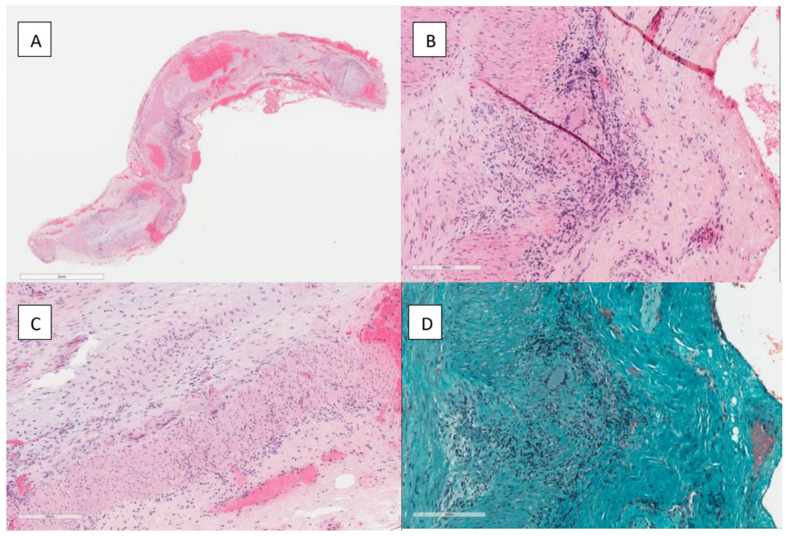
(**A**) Fragmentation of the elastic fibers of the artery wall can be appreciated (hematoxylin eosin stain; magnification 200×). (**B**) Biopsy of the temporal artery showing patchy phlogosis in the artery wall (hematoxylin eosin stain; low magnification). (**C**) At higher magnifications, a florid granulomatous inflammation with lymphocytes and giant cells was visible (hematoxylin eosin stain; magnification 200×). (**D**) Masson’s trichrome stain showing florid granulomatous phlogosis of the artery wall, with lymphocytes and giant cells (magnification 200×).

In July 2021, one month after the onset of symptoms, he presented to the Emergency Department of *A.O.U. Città della Salute e della Scienza* Hospital, due to the persistence of symptoms; the laboratory tests showed the persistent elevation of CRP and leukocytosis. Head and neck ultrasounds with Doppler did not show temporal artery morphology alterations nor blood flow anomalies, and the rheumatological examination was negative; in particular, no signs of synovitis were present and the patient did not report pain and stiffness in the neck and pelvic girdle. Suspecting large vessel vasculitis, temporal artery biopsy was performed; the histopathologic findings demonstrated transmural inflammatory infiltrate with giant cells, compatible with giant cell arteritis (Figure 1).

**Figure 2:**
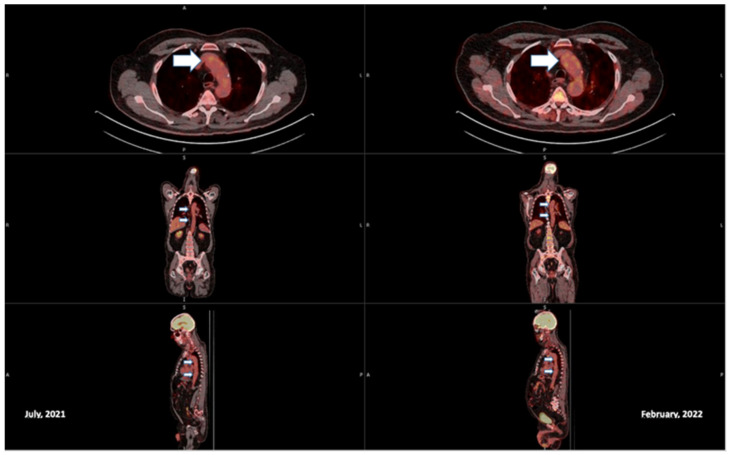
Baseline and fused PET/CT transaxial, coronal and sagittal images; arrows indicate aortic uptake.

**Figure 3:**
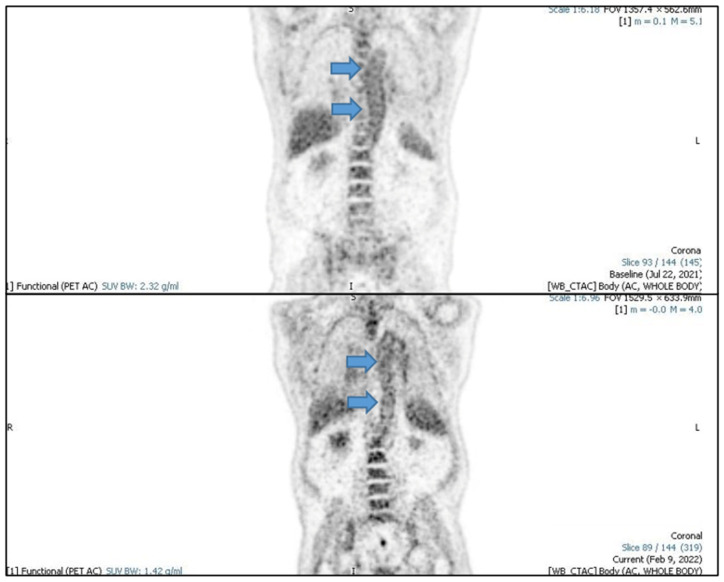
PET coronal images, arrows indicate aortic uptake.

**Figure 4:**
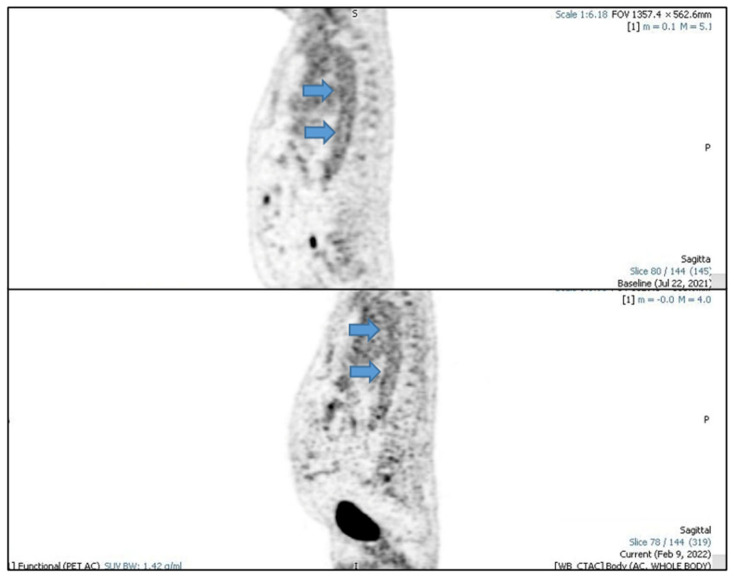
PET sagittal images; arrows indicate aortic uptake.

Furthermore, a fluorine-18-fluorodeoxyglucose positron emission tomography scan (FDG-CT PET) showed evidence of active large vessel vasculitis with abnormal artery uptake at the level of the ascending aorta, aortic arch (SUV max 4.2), descending aorta, iliac axes bilaterally and subclavian bilaterally (Figure 2, Figure 3 and Figure 4).

Oral corticosteroid therapy was promptly instituted using prednisone 1 mg/kg at a single daily dose for 4 weeks and subsequent slow tapering over 8 months to a dosage of 5 mg/day, alternating 7.5 mg/day. Prophylaxis with isoniazid was also introduced due to a positive Quantiferon-TB test. Two months after starting prednisone, after collegial discussion with dermatologists, Tocilizumab was also introduced. After a few weeks, the CRP value normalized and the headache, fatigue, jaw claudication, scotomas, pharyngodynia and dry cough disappeared. In February 2022 a fluorine-18- fluorodeoxyglucose positron emission tomography scan was performed that showed the persistence of a very modest fixation of the walls of the great vessels, in particular at the level of the whole aorta (SUV max. 3.8), of the iliac axes and of the subclavian arteries, with an intensity comparable to the hepatic background (SUV max. 3.9) (Figure 2, Figure 3 and Figure 4).

## 3. Discussion

Skin manifestations, including cutaneous vasculitis and vasculopathy were reported after the coronavirus disease 2019 (COVID-19) and different pathogenetic mechanisms have been hypothesized. Patients with robust type I interferon response could develop during the infection, lymphocytic perivascular cuffing, leading to chilblain-like lesions. The virus can also cause immune complex deposition, complement activation and neutrophil recruitment. A similar mechanism involving the anaphylatoxins and inducing excessive mastocyte activation was hypothesized on the basis of the development of urticarial vasculitis. Some patients developed severe COVID-19, characterized by indirect endothelial injury, due to elevated proinflammatory cytokines and ischemia-induced complement activation [9].

A recent survey, including 1377 patients affected by rheumatic diseases vaccinated with two dose of SARS-CoV-2 mRNA-based vaccines reported flares of their underlying disease, requiring treatment in 11% of participants. Participants described systemic events, such as myalgia and fatigue, with a higher frequency than reported in clinical trials; similarly to the trials, reactogenicity was increased after the second dose. In most cases local and systemic reactions did not interfere with daily activity—only one patient reported hospitalization for the management of a systemic event. Authors suggested an immunologic priming due to the positive association between a prior SARS-CoV-2 infection and flare. This study has some limitations, including a lack of data on immunomodulatory timing and dosing and baseline disease activity and prior rate of flare; furthermore, the data are also susceptible to responder bias [1].

Moreover, Ursini et al. recently published a case series of short-term inflammatory musculoskeletal manifestations after COVID-19 vaccine administration, from the “COVID-19 and Autoimmune Systemic Diseases”, an Italian new network of rheumatologists, equally distributed across the country, created during the COVID-19 pandemic with the aim of improving knowledge about COVID-19 and rheumatic diseases by providing real-life data obtained from participating centers. This report included 66 patients with onset of inflammatory musculoskeletal manifestations within 4 weeks of COVID-19 vaccine administration. The most common clinical presentation was characterized by shoulder girdle and hip girdle pain and stiffness with acute-phase reactant elevation resembling polymyalgia rheumatica (PMR-like) in 41% of patients, followed by oligoarthritis (32%) and polyarthritis (27%). Only two patients presented inflammatory back pain and an MRI highlighted active sacroiliitis and/or spondylitis; 50–78% of patients were treated with glucocorticoids. Five patients were treated with polyarthritis, five with oligoarthritis and three with PMR-like presentations were treated with disease-modifying antirheumatic drugs. Although a clear cause–effect relationship has not been demonstrated, these data suggest that inflammatory musculoskeletal symptoms could occasionally occur shortly after the administration of the COVID-19 vaccine. The authors highlight that the benefits of vaccination outweigh the slightest risks associated with these rare inflammatory complications, likely reflecting a transient reactogenic response to the vaccine [10].

Severe acute respiratory syndrome coronavirus 2 (SARS-CoV-2) infection has caused several complications in the past years, even if vaccines have been burdened with local or systemic side effects. Local reactions include erythema, tenderness, induration at the injection site, while systemic adverse events include fever, chills, headache, cough, coryza and, rarely, allergic reactions. However, rare manifestations with involvement of various organs such as renal, ocular, dermatologic, gastrointestinal, hematologic, lymphatics, cardiovascular and neurologic systems have also been described [11].

There have been rare reports of IgA vasculitis reactivation, following the COVID-19 vaccination [12,13,14]; IgA vasculitis may involve skin, joints, intestines and the kidneys’ small blood vessels and its pathogenesis is yet to be understood completely, even if the role of genetic and environmental factors and infections has been suggested [15]. Similarly, some cases of leukocytoclastic vasculitis following COVID-19 mRNA-based vaccines have been reported. In the study by Sollini et al., 18F-FDG PET/CT findings hypothesized that post-COVID-19 vasculitis could be considered a cause of prolonged symptoms (fatigue, dyspnea and chest pain) that have been reported after COVID-19 recovery [16]. Urticarial vasculitis (UV) was reported both as a complication and as an adverse event of vaccination [17,18]. UV is an inflammatory skin disease characterized by urticarial rash lasting more than 24 h, which typically has dyschromic results. A patient with UV must undergo a careful differential diagnosis, even if idiopathic remains the most common form, as UV also has been described in several reports with onset after COVID-19 infection and vaccination.

Dash et al. reported a case of a 27-year-old who developed multiple erythematous urticarial plaques over their torso and extremities, fever and bilateral knee and ankle joint pain and elevated C-reactive protein after the second dose of SARS-CoV-2 vaccine. Histopathology revealed the disruption of the walls of the small vessels, endothelial swelling, erythrocyte extravasation and mild perivascular infiltration comprising neutrophils, eosinophils and lymphocytes, compatible with urticaria vasculitis diagnosis [18].

Cutaneous vasculitis is an inflammatory dermal blood vessel disorder that may involve only the skin or may be the expression of systemic vasculitis. Both IgA vasculitis, UV and cutaneous diagnosis is often based on skin biopsy [19]. Although the exact incidence of cutaneous vasculitis following the COVID-19 vaccine has not been determined, Uh et al. has presented five cases of cutaneous small-vessel vasculitis following the ChAdOx1-S COVID-19 vaccine [20].

Rheumatoid arthritis flares have been reported after COVID-19 vaccination [21] and, in the past, following the tetanus, rubella, hepatitis B and influenza vaccines [22]; the cause may be attributed to the molecular mimicry. It is noteworthy that rheumatoid arthritis flare-ups after COVID-19 infection have also been reported [23].

Furthermore, other immunological manifestations, such as IgA nephropathy and subacute thyroiditis, have been similarly reported after COVID-19 mRNA vaccines [11].

Gillion et al. published the case of a 77-year-old man who developed medium vessel vasculitis associated with acute granulomatous nephritis, after the first dose of the ChAdOx1-S vaccine. Four weeks after the vaccinations, the patient presented with constitutional symptoms, such as anorexia, fever, night sweats and increased C-reactive protein level; a fluorine-18-fluorodeoxyglucose positron emission tomography scan showed the diffuse hypermetabolism of medium vessels, and a kidney biopsy revealed diffuse interstitial edema with non-caseating, non-necrotizing granulomas around small vessels, without immune deposits. Within 4 weeks after treatment with methylprednisolone serum, creatinine and C-reactive protein normalized. Although causality cannot be proven, the plausibility of these associations is supported by the timing and the absence of other causes [24,25].

Furthermore, although the pathogenesis of systemic vasculitis is still not fully understood, in the past the association of vasculitis onset with the administration of vaccines against influenza and pertussis has been hypothesized [8]. Sporadic cases of giant cell arteritis secondary to COVID-19 vaccination have recently been reported by the French Pharmacovigilance Network, although vaccine causality has not yet been assessed. In a recent study based on pharmacovigilance statistical analyses of spontaneous reports on VigiBase, the WHO global case safety report database observed 147 GCA cases, 290 PMR cases and 9 GCA with PMR cases occurring after COVID-19 vaccines; however, when comparing these data with the use of influenza vaccines, no increased reporting was found (giant cell arteritis ROR 0.5, 95% CI: 0.4, 0.7 and polymyalgia rheumatica ROR 0.2, 95% CI: 0.2, 0.2). Although this study has the limit of data based on spontaneous reports, these results support a safety signal [26].

Recently, a case report of an 83-year-old woman with GCA onset 24 h after the COVID-19 mRNA vaccine (BioNTech/Pfizer) was published; she presented to the emergency department with a 2-week history of disruptive cervical pain, headache, scalp tenderness and increased inflammatory markers, without jaw claudication, visual manifestations or polymyalgia rheumatica symptoms, and was treated successfully with steroids and methotrexate. Authors suggested that the new technologies used to create vaccines make them safer and faster to develop but utilize the same mechanisms that cause antigen cross-reactivity. Moreover, it has been speculated that infectious agents may play a role in giant cell arteritis pathophysiology; this hypothesis is due to the seasonal incidence of this vasculitis and the presence of viral antigens on temporal artery biopsies in some case reports [2].

Most anti-SARS-CoV-2 vaccine-associated cutaneous vasculitis is thought to have an immune-complex deposition with complement activation pathophysiology. This pathophysiology is also thought to be responsible for the development of vasculopathy and vasculitis. A pro-inflammatory state may be induced by SARS-CoV-2 vaccine components that structurally resemble host proteins. This pro-inflammatory state is then followed by the activation of autoreactive B and T cells, the production of antibodies and the subsequent immune complete deposition in the small vessels of the skin, with the potential involvement of internal organs. The phenomenon of molecular mimicry might also be important. Due to the vaccine’s immune-enhancing characteristics, it is also important to take into account an unrelated antigen or a hereditary predisposition that has become apparent [11]. Although the overall incidence of vaccine-triggered autoimmunity is low, vaccination should continue as planned, but rheumatologists and clinical immunologist worldwide should be aware of autoimmune diseases as a new potential adverse event of SARS-CoV-2 vaccines.

## 4. Conclusions

In conclusion, even though it can be challenging to establish a direct cause-and-effect connection, receiving the COVID-19 vaccine can occasionally be closely correlated with the onset of inflammatory musculoskeletal symptoms. Despite numerous case reports linking vasculitis and COVD-19 vaccination, it is there a paucity of studies addressing the pathophysiology of these demonstrations. Nevertheless, the advantages of the COVID-19 vaccine outweigh this uncommon potential risk.

## Data Availability

Data are available upon request.
